# Lack of Visual Experience Affects Multimodal Language Production: Evidence From Congenitally Blind and Sighted People

**DOI:** 10.1111/cogs.13228

**Published:** 2023-01-06

**Authors:** Ezgi Mamus, Laura J. Speed, Lilia Rissman, Asifa Majid, Aslı Özyürek

**Affiliations:** ^1^ Centre for Language Studies Radboud University; ^2^ Max Planck Institute for Psycholinguistics; ^3^ Department of Psychology University of Wisconsin – Madison; ^4^ Department of Experimental Psychology University of Oxford; ^5^ Donders Center for Cognition Radboud University

**Keywords:** Blindness, Spatial language, Auditory perception, Motion events, Co‐speech gesture, Pointing

## Abstract

The human experience is shaped by information from different perceptual channels, but it is still debated whether and how differential experience influences language use. To address this, we compared congenitally blind, blindfolded, and sighted people's descriptions of the same motion events experienced auditorily by all participants (i.e., via sound alone) and conveyed in speech and gesture. Comparison of blind and sighted participants to blindfolded participants helped us disentangle the effects of a lifetime experience of being blind versus the task‐specific effects of experiencing a motion event by sound alone. Compared to sighted people, blind people's speech focused more on path and less on manner of motion, and encoded paths in a more segmented fashion using more landmarks and path verbs. Gestures followed the speech, such that blind people pointed to landmarks more and depicted manner less than sighted people. This suggests that visual experience affects how people express spatial events in the multimodal language and that blindness may enhance sensitivity to paths of motion due to changes in event construal. These findings have implications for the claims that language processes are deeply rooted in our sensory experiences.

## 1 Introduction

We experience the world through multiple perceptual channels, such as hearing footsteps while watching someone running upstairs. We also express our multimodal experience in language using different modalities, as in speech and gesture. Modern theories of language and cognition, including multimodal language theories, differ in whether they view language as a relatively embodied or disembodied system (see Meteyard et al., 2012, for a review). According to embodied theories, language processes—both speech and gesture—are deeply rooted in sensory and motor experience (e.g., Barsalou, 2016; Hostetter & Alibali, 2008, 2019; Pouw et al., 2014; Pulvermüller, 2013; Wilson, 2002), whereas disembodied symbolic theories suggest that language processing relies on abstract, modality independent representations instead, which interface with perceptual representations later during semantic processing (e.g., Levelt, 1989; Mahon & Caramazza, 2008; Patterson et al., 2007).

Congenitally blind people, who do not have a typical visual experience, provide an interesting opportunity to explore the relationship between multimodal experience and language. While some studies have claimed lack of visual experience does not change the way blind people understand and use language (e.g., Kim et al., 2021; Landau & Gleitman, 1985; Mahon et al., 2009; Özçalışkan et al., 2016, 2018), there is also evidence to the contrary (e.g., Connolly et al., 2007; Iverson, 1999; Iverson & Goldin‐Meadow, 1997; Shepard & Cooper, 1992). Thus, there is an ongoing debate over whether and how experience shapes language (e.g., Barsalou, 2016; Bedny & Saxe, 2012; Mahon & Caramazza, 2008).

On the one hand, 3‐year‐old blind children understand the semantics of vision‐related words—such as look and see—in a manner comparable to their sighted peers (Elli et al., 2021; Landau & Gleitman, 1985). Studies on word comprehension also show no difference between blind and sighted people in semantic judgments of object concepts, actions, and vision‐related terms (Bedny et al., 2012, 2019; Kim et al., 2021; Mahon et al., 2009; Marmor, 1978; Saysani et al., 2021). Similarly, previous studies of spatial language have emphasized the similarities in language between blind and typically sighted people. For example, in one study of congenitally blind, sighted, and blindfolded speakers of Turkish and English, participants explored static scenes depicting motion with figurines—e.g., dolls in different postures so as to indicate running (Özçalışkan et al., 2016). Both blind and blindfolded participants explored scenes haptically, while sighted people explored them visually. All speakers described motion events in speech and co‐speech gesture according to the typology of their language. So, Turkish speakers were more likely to mention path (i.e., the trajectory of movement) and manner (i.e., how the movement was performed) in separate clauses (e.g., koşarak eve geldi “she came to the house running”), whereas English speakers conflated these components into one clause (e.g., she ran to the house). Critically, gestures followed the language‐specific patterns regardless of whether people were blind, blindfolded, or sighted. This suggests that visual experience plays little role in language use.

On the other hand, there is evidence that there may be differences in language knowledge and use between blind and sighted people (e.g., Connolly et al., 2007; Iverson, 1999; Iverson & Goldin‐Meadow, 1997; Kim et al., 2019; Lenci et al., 2013; Shepard & Cooper, 1992). This holds for spatial language too. For example, English‐speaking blind and sighted people differ in their descriptions of routes in speech and gesture—especially regarding path expressions (Iverson, 1999; Iverson & Goldin‐Meadow, 1997). When describing a familiar route in their school, blind children segmented the path according to several landmarks, whereas sighted and blindfolded children described paths more holistically using fewer landmarks and with more gestures accompanying speech (Iverson, 1999; Iverson & Goldin‐Meadow, 1997). So, a blind child described a route as: “*Turn left, walk north, then you'll see the office, then you'll see 106, then 108, then 110, 112, then there's a doorway. Then there's a hall…*,” whereas a sighted child said: “*when you get near the staircase you turn to the left*” (p. 463). Compared to gesture, speech is better suited to represent sequential information coming from auditory and haptic input. Since gesture does not require linearization to the same degree that speech does, it has been described as conveying meaning in a more “holistic” manner that is through analog, iconic, and gradient representations (McNeill, 1992; McNeill & Duncan, 2000). This theory led Iverson and Goldin‐Meadow (1997) to suggest that gesture is better suited for holistic than segmented meaning elements since gesture as a visual format, by nature, is not well‐suited for linearization. Accordingly, they found that gesture frequency decreases with segmented path descriptions (i.e., “when path is broken up into a series of locations” on p. 463, Iverson and Goldin‐Meadow, 1997), particularly when the spatial layout is large‐scale and includes multiple paths (Iverson, 1999). This is corroboratory evidence from language that spatial cognition in blind people is more sequential than in sighted people (e.g., Cattaneo & Vecchi, 2011; Iachini et al., 2014; Noordzij et al., 2006; Pasqualotto & Proulx, 2012; Ruggiero et al., 2021; Thinus‐Blanc & Gaunet, 1997; Vercillo et al., 2018), and lack of visual experience may shape spatial language via altered spatial representations.

In light of these conflicting results, it is unclear what role the visual experience plays in multimodal spatial language use. The previous studies, while informative, have some potential drawbacks which make them difficult to synthesize. First, some of these studies examined pre‐existing spatial representations—i.e., familiar routes (Iverson, 1999; Iverson & Goldin‐Meadow, 1997), whereas others used novel spatial scenes (Iverson, 1999; Özçalışkan et al., 2016, 2018). Second, some studies did not control the type of input at encoding—i.e., how participants learned routes (Iverson, 1999; Iverson & Goldin‐Meadow, 1997), and some did not equate input modalities—i.e., sighted participants explored scenes visually, whereas blind and blindfolded participants explored scenes haptically (Iverson, 1999; Özçalışkan et al., 2016, 2018). In addition, in Özçalışkan et al. (2016, 2018), time spent exploring scenes visually versus haptically was not controlled, so haptic groups could have taken longer exploring scenes which allowed them to compensate for differential input. Finally, in some studies, speakers were explicitly asked to gesture as they described scenes (Özçalışkan et al., 2016, 2018), which might have affected how scenes were encoded.

### 1.1 The present study

The present study mitigates these limitations by conducting a new experiment with blind and sighted people where all participants receive the same motion event input. Auditory motion events were recorded depicting a person walking, running, or limping to and from landmarks and presented to participants to elicit verbal descriptions and spontaneous co‐speech gesture. Our study has the advantage that it includes ecologically relevant stimuli. Hearing sounds of human locomotion is familiar to both blind and sighted people, and previous research has shown that sighted people are able to extract information about path and manner of motion from auditory input alone (Geangu et al., 2021; Mamus et al., 2019, 2022). To better distinguish whether potential differences in the linguistic encoding of spatial information arise from the long‐term effect of blindness or are due instead to momentary effects of lack of vision at encoding, we compared blind and sighted people to blindfolded people. It has been shown that closing the eyes while attending to auditory information modulates attention (Wöstmann et al., 2020). By comparing blindfolded to blind participants, we are better able to determine whether any differences between sighted and blind people reside in momentary stimulus affordances.

We had different predictions concerning speech and gesture based on slightly different literatures regarding perceptual language and current theories of multimodal language production. Accordingly, we will consider the predictions regarding speech and gesture in turn.

#### 1.1.1 Speech

A number of studies report that vision dominates in the perceptual lexicons of languages (e.g., Floyd et al., 2018; Levinson & Majid, 2014; Majid et al., 2018; San Roque et al., 2015; Viberg, 1983; Winter et al., 2018) and leads to richer motion event descriptions (more manner distinctions encoded) than auditory information alone (Mamus et al., 2022). Together, this suggests that descriptions produced by blind people may be different compared to sighted people. Specifically, we predicted that blind people may produce fewer motion event descriptions overall than sighted people. At the same time, blind people are known to rely more extensively on audition than sighted people to localize space, and are often better than sighted people at processing auditory information (e.g., Battal et al., 2020; Gougoux et al., 2004; Röder et al., 1999; Wan et al., 2010). So, blind participants might provide as many motion event descriptions—if not more—than sighted participants.

In addition to the overall number of motion event descriptions, we examined speech for landmark use when participants expressed paths. Earlier route description studies found that blind people segment path descriptions using landmarks more than sighted people (Iverson, 1999; Iverson & Goldin‐Meadow, 1997). Here, we test if this hypothesis is confirmed with experimentally controlled motion events and examine whether blind participants still use more landmarks than blindfolded and sighted participants.

Furthermore, previous spatial cognition studies have found that blind people rely mainly on an egocentric rather than allocentric spatial frame of reference (e.g., Cattaneo & Vecchi, 2011; Iachini et al., 2014; Pasqualotto & Proulx, 2012). Accordingly, we predict that spatial locations will be described more in relation to blind people's own position in space. That is, blind people may mention landmarks in relation to their own body (i.e., self‐anchored; *from my left*), instead of using external coordinates (e.g., object‐anchored; *from the elevator*). Therefore, we also tested whether mentions of landmarks in the blind participants were primarily self‐anchored and those of non‐blind participants were more object‐anchored.

Finally, we examined speech for the encoding of path and manner separately. With regard to path, based on the previously attested differences in the encoding of path (i.e., segmented paths with more landmarks in blind vs. non‐blind; Iverson, 1999; Iverson & Goldin‐Meadow, 1997), it might be expected that increased segmentation would increase the use of path verbs. So, blind participants may mention path more often within each description in speech. For manner, vision seems to provide richer information about manner than audition (Malt et al., 2014; Mamus et al., 2022), so perhaps blind participants will produce fewer manner expressions. On the other hand, earlier studies suggest that blind people can differentiate the semantic similarity of actions as well as sighted people (Bedny et al., 2012, 2019), so perhaps there will be no difference between groups.

#### 1.1.2 Gesture

Theories vary in their specification of the interaction between speech and gesture, as well as in how they view the nature of spatial imagery underlying gesture production (de Ruiter, 2000, 2007; Hostetter & Alibali, 2008; Kita & Özyürek, 2003; Krauss et al., 2000; McNeill, 1992; McNeill & Duncan, 2000). Gesture theories typically emphasize the role of visuo‐spatial imagery in gesture production (e.g., de Ruiter, 2000; Hostetter & Alibali, 2008; Kita & Özyürek, 2003; Krauss et al., 2000), although studies have shown that gesture can be derived from auditory information alone in sighted people too (Holler et al., 2022; Mamus et al., 2022). Though, if visuo‐spatial imagery is one of the main sources of gesture production, the lack of any visual experience, as in the case of congenital blindness, might lead to differences in how people gesture in relation to spatial events. Indeed, earlier studies found the rate of spontaneous gesturing was lower among blind than sighted people when describing routes (Iverson, 1999; Iverson & Goldin‐Meadow, 1997) and motion events (Özçalışkan et al., 2016, 2018). Based on this, we predicted fewer spontaneous gestures among blind than non‐blind people in motion event descriptions.

Second, we examined speakers’ pointing gestures used with mentions of landmarks in speech. Pointing gestures can be used to direct attention to an object or place an object in gesture space during communication (e.g., McNeill, 2000). While describing a motion event, speakers can use pointing gestures to locate landmarks to be communicatively clear. We know blind people are good at localizing sounds and often outperform sighted people (e.g., Battal et al., 2020; Lessard et al., 1998; Röder et al., 1999; Voss et al., 2004). So, it might be expected that blind participants would produce more pointing gestures than non‐blind participants.

Finally, we examined speakers’ iconic gestures for path and manner. Previous studies (Iverson, 1999; Iverson & Goldin‐Meadow, 1997) claimed that gesture production decreases with segmented speech because gestures are better suited for holistic expression due to their visual format being less suited for linearization than speech (McNeill, 1992; McNeill & Duncan, 2000). Based on this, if blind participants use more path verbs to segment their descriptions than non‐blind participants, we might not expect a similar increase in the frequency of path gestures in blind compared to non‐blind participants. But, according to speech–gesture interface theories, one would also expect gestures to parallel speech patterns and align with speech frequency (e.g., Kita & Özyürek, 2003; Özçalışkan et al., 2016, 2018). If so, there would be more path gestures in blind than non‐blind participants. Similarly, for manner gestures, visual experience of human locomotion may be necessary to map the sounds of manner into gesture regardless of speech. If so, blind participants would express manner less often in gesture than non‐blind participants. Alternatively, gesture patterns may align with speech and so, if blind participants mention manner in their speech at comparable rates to non‐blind participants, we would not expect a difference in manner gestures.

## 2 Method

### 2.1 Participants

Twenty‐one congenitally blind (*M* = 28.19 years, *SD* = 6.56, range = 18–40), 21 blindfolded (*M* = 27.43 years, *SD* = 6.10, range = 19–49), and 21 sighted (*M* = 27.29 years, *SD* = 6.61, range = 20–41) native Turkish speakers were paid to participate in the experiment. The sample size was determined by access to the special population with the control groups matched to the number of blind participants recruited. At the time of testing, 12 blind participants had light perception and nine had total blindness (see Table 1 for detailed characteristics of the blind participants). Blindfolded and sighted participants with normal or corrected‐to‐normal vision were matched for age, gender, and education to blind participants. Participants were tested in a quiet room on the Boğaziçi University campus. They all were paid the equivalent of €9 in Turkish Lira for their participation and provided written informed consent approved by the IRB committees of Boğaziçi and Radboud Universities.

**Table 1:** Blind participants demographic information

Ss	Gender	Age	Age of blindness	Cause of blindness	Residual light perception^a^	Highest level of education
101	NB	25	Birth	Retinal degeneration	Yes	BA (student)
102	F	26	Birth	Retinal degeneration	Yes	BA
103	M	19	Birth	Optic nerve atrophy	Yes	BA (student)
104	M	25	Birth	Retinitis pigmentosa	Yes	BA
105	M	24	Birth	Optic nerve hypoplasia	Yes	BA
106	F	25	Birth	Retinitis pigmentosa	Yes	BA (student)
108	F	28	Birth	Retinitis pigmentosa	Yes	BA
109	F	25	Birth	Optic nerve hypoplasia	Yes	BA
112	M	25	7 months	Retinoblastoma	None	BA (student)
114	M	20	Birth	Premature birth	None	BA (student)
115	M	40	Birth	Hereditary/ unknown cause	Yes	PhD
116	M	26	Birth	A genetic disease	Yes	MA
117	M	24	Birth	Anophthalmia	None	BA
118	M	36	Birth	Retinitis pigmentosa	None	BA
119	M	30	Birth	Dry optic nerves	None	BA
120	M	33	Birth	Norrie disease	None	BA (student)
122	F	39	Birth	Hereditary/ unknown cause	Yes	MA
123	F	39	6 months	Retinoblastoma	None	MA (student)
125	M	35	6 months	Retinoblastoma	None	BA
126	F	18	Birth	Premature birth/retinal tear	None	BA (student)
127	F	30	Birth	Optic nerve atrophy	Yes	MA

^a^ Under optimal conditions.

### 2.2 Auditory stimuli

We audio‐recorded locomotion and non‐locomotion events performed by an actress. Locomotion events were the critical items and non‐locomotion events were filler items. We created 12 locomotion events by crossing three manners (walk, run, and limp) with four paths (to, from, into, and out of) in relation to a landmark object (door or elevator)—e.g., “*someone walks from a door*.” An audio recorder was placed next to the landmark objects. For *to* and *into* events, the actress approached the landmarks, so the path direction approaching the audio recorder—and for *from* and *out of* events, the actress moved away from the landmarks, so the path direction moving away from the audio recorder. To ensure that landmark objects were recognizable, we created auditory landmarks. For example, for the “elevator” landmark, we recorded the sound of an elevator ring—the tone that is heard when an elevator arrives at its destination. We also recorded the sound of an elevator door opening automatically. Then, we created a combined audio file: the ring (representing the arrival of the elevator) followed by the opening sound.

**Fig. 1:**
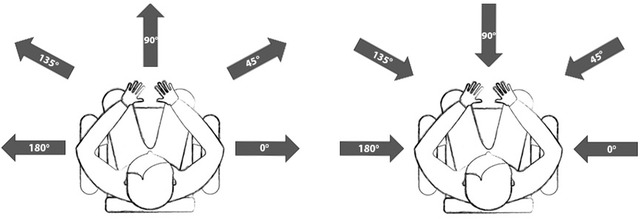
Five movement angles for “*from*” and “*out of*” events (left) and “*to*” and “*into*” events (right). The figure was taken from Mamus et al. (2019).

In addition, we edited the path azimuth angle using Soundtrack Pro audio editing software to vary the path motion. Five movement angles were created in a semicircular space ranging from 90° left to 90° right with 45° intervals. From the right to the left, these were: 0° (right), 45° (right‐sided), 90° (front), 135° (left‐sided), and 180° (left) motions (Fig. 1). We created 12 events with five movement angles, resulting in 60 events in total. All locomotion events were exported as 5.1 surround sound.

To create non‐locomotion events, the same actress performed “transitive” actions with different objects (e.g., opening a can and chopping a cucumber), and audio was recorded at a fixed distance. We do not examine these items further.

There were 77 trials per person, including a total of 60 locomotion events and 17 non‐locomotion events. Locomotion events lasted on average 9 s (*SD* = 1.9) and non‐locomotion events 8 s (*SD* = 2.2). The event list and stimuli are available at https://osf.io/qsr7j/.

### 2.3 Procedure

The procedure was the same for all groups, except that blindfolded participants’ eyes were covered with a mask before they entered the room. Five speakers were placed 1.34 m from the participant's head and approximately 95 cm from the ground in a 5+1 surround system configuration. Front left and right speakers were placed 30° off center, and rear left and right speakers were 110° off center. Participants sat in the middle of the speakers. The experimenter stayed in the room to initiate the task and advance trials on a laptop using Presentation Software.

Events were presented aurally and participants were asked to describe each event at their own pace without any instructions about gesture use. They were told that another participant would watch the video recording of their descriptions and listen to the same events to match descriptions with events. At the beginning of the experiment, participants performed two practice trials consisting of one locomotion and one non‐locomotion event. Further clarification was provided, if necessary, after the practice trials. Descriptions were recorded with two video cameras. One camera was approximately 1.5 m across from the participants and the other recorded the top view of the participants’ frontal space so as to capture arm and hand movements. Participants filled out a demographic questionnaire—including questions about blindness history for blind participants—on another laptop after the event description task. The experiment lasted around 45 min.

### 2.4 Coding

#### 2.4.1 Speech

Descriptions of locomotion and non‐locomotion events were annotated by native Turkish speakers using ELAN (Wittenburg et al., 2006), but only descriptions for the locomotion events were transcribed and coded. Event descriptions were split into sentence units, defined as a verb and its associated arguments (Azar et al., 2020; Özçalışkan et al., 2016). Sentence units could contain a subordinate clause as well. Sentence units were then coded as motion event descriptions if they referred to locomotion (e.g., someone is running into an elevator); sentence units including a transitive event, e.g., “*opening a door*” or “*ringing a bell*,” or other information, e.g., “*wearing high heels*” or “*a wooden floor*,” were coded as irrelevant to the target event.

Motion event descriptions were coded for: landmark—either source (start point of movement) or goal (end point of movement), (b) path (trajectory of motion), and (c) manner (how the action is performed). We also coded whether landmarks reference either: (i) external objects (e.g., from/to a door or elevator) or (ii) self‐anchored (the speaker's body, e.g., to/from my left)—see Table 2 for an example. We calculated the interclass correlation coefficient (ICC) between two coders to measure the strength of inter‐coder agreement for landmark, path, and manner in speech (Koo & Li, 2016). Agreement between coders was .94 for object‐anchored landmark, .96 for self‐anchored landmark, .98 for path, and .95 for manner of motion.

**Table 2:** An illustrative example of a description and its coding

Turkish description	Asansör	‐den	çık	‐ıp	sağ	‐ım	‐a	doğru	yürü	‐yor
Glossing	elevator	ABL	exit	GER	right	1sPOSS	DAT	towards	walk	PRS.3SG
Turkish description	object‐anchored landmark		path		self‐anchored landmark				manner	
English translation	“(someone) exited from the elevator walking towards my right”									

#### 2.4.2 Co‐speech gesture

**Fig. 2:**
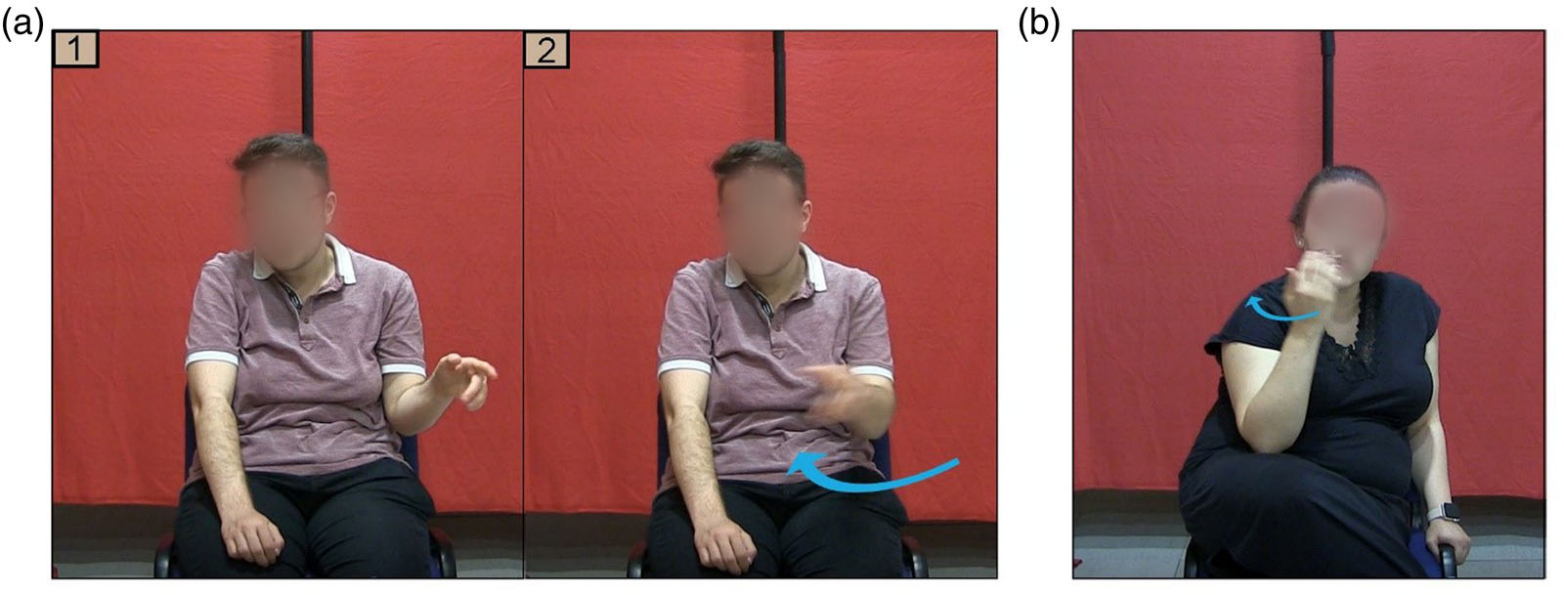
(a) A blind participant produces a pointing gesture (1) to a landmark and (2) then a path gesture while saying *soldan sağa doğru geldi* “came from the left towards the right.” (b) A sighted participant produces a path gesture (hand moving backward) while saying *içeri giriyor* “entering inside.”

Participants’ spontaneous representational gestures (pointing and iconic) were identified for each target motion event description (Kita, 2000). We coded gesture strokes (i.e., the meaningful phase of a gesture) that co‐occurred with parts of the description. Each continuous instance of hand movement was coded as a single gesture. Pointing gestures were either head or hand‐pointing gestures to empty locations in gesture space and were coded when they represented a source/goal landmark in speech. For example, if a speaker pointed to a spatial location to indicate the starting point of movement without showing its trajectory, the gesture was coded as a pointing gesture referring to the localization of a landmark (e.g., Fig. 2). Iconic gestures representing trajectory or manner of motion were further classified into the following categories:

- ***path‐only*** gestures depict the trajectory of movement without representing manner
- ***manner‐only*** gestures show the style of movement without representing trajectory
- ***path+manner*** gestures depict both trajectory and manner of motion simultaneously

We calculated the ICC between two coders to measure the strength of inter‐coder agreement for identifying a gesture and coding each type of gesture. Agreement between coders was .88 for identifying gestures and between .82–.93 for type of gesture—i.e., .89 for coding pointing gestures, .89 for coding path only, .93 for manner only, and .82 for path+manner gestures.

## 3 Results

To analyze the data, we used linear mixed‐effects regression models (Baayen et al., 2008) with random intercepts for participants and items, using the packages *lme4* (Version 1.1–28; Bates et al., 2015) with the optimizer *nloptwrap* and *lmerTest* (Version 3.1–3; Kuznetsova et al., 2017) to retrieve *p*‐values in R (Version 4.1.3; R Core Team, 2022). We conducted linear mixed‐effects models on the different motion elements in speech and gesture. To assess the statistical significance of the fixed factors and their interaction, we used likelihood‐ratio tests with *χ* ^2^, comparing models with and without the factors and interaction of interest. For post‐hoc comparisons and to follow‐up interactions, we used *emmeans* (Version 1.7.3; Lenth, 2022). Data and analysis code are available at https://osf.io/qsr7j/.

### 3.1 Speech

We examined speech for the overall amount of motion event descriptions, landmark use, and reference to path and manner.

#### 3.1.1 Overall amount of motion descriptions

First, we tested whether participants differed in the speech they produced for motion events. We ran a glmer model with the fixed factor of group (blind, blindfolded, or sighted) on binary values for mention of motion event description in speech (0 = no, 1 = yes) as a dependent variable. It revealed no effect of group on motion event description, *χ* ^2^ (2) = .91, *p* = .635.

#### 3.1.2 Landmark use in speech

**Fig. 3:**
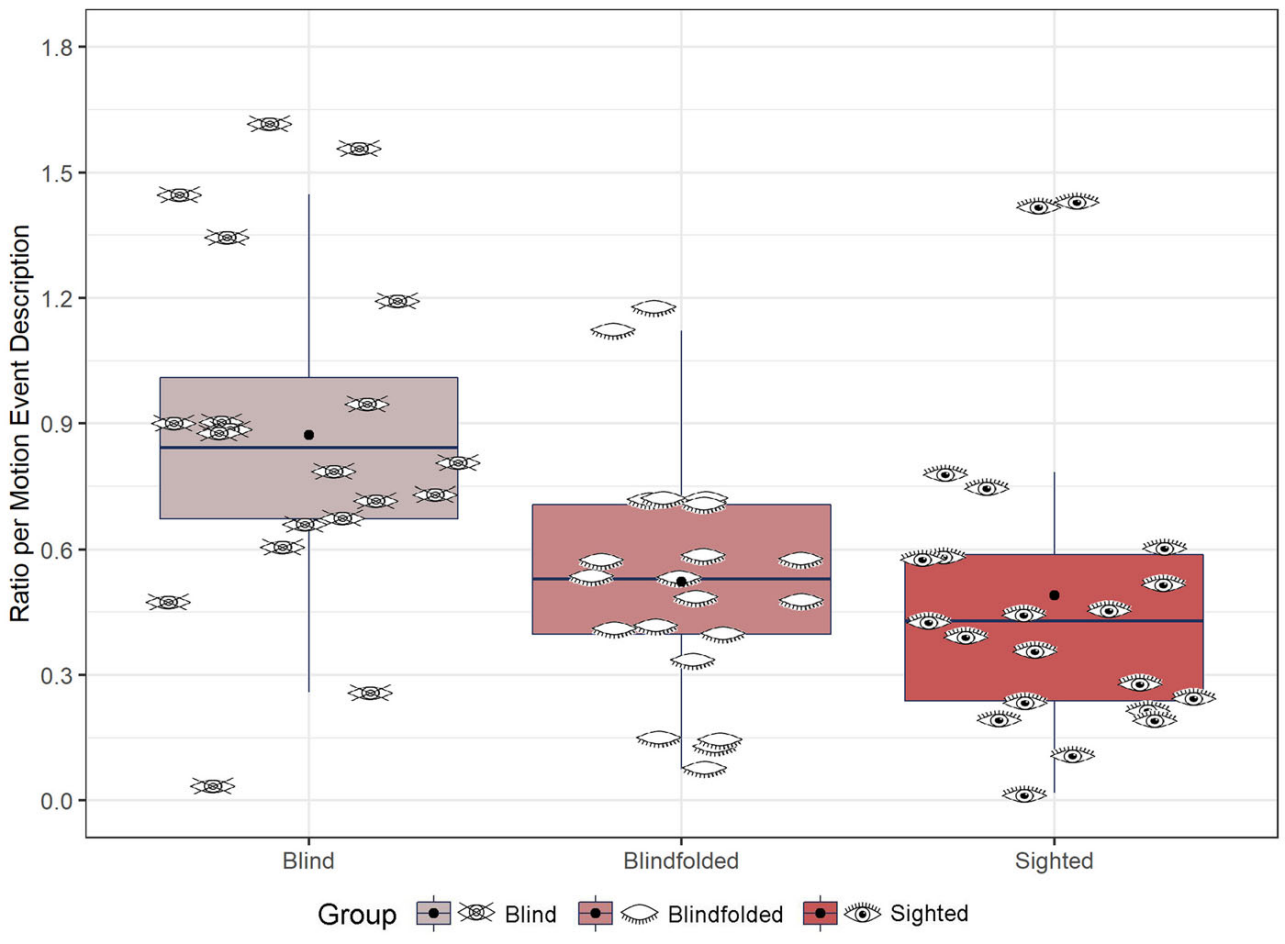
Overall landmarks in speech. Eye icons represent the average ratio for each participant. Black dots represent the group mean.

We predicted that blind participants would segment descriptions using more mention of landmarks than blindfolded and sighted participants. To account for baseline differences in the number of motion event descriptions produced, we calculated the ratio of landmark (including all types of landmark) per motion event description for each participant and item. We ran an lmer model with the fixed factor of group using the ratio of mention of landmark per motion event description as the dependent variable (Fig. 3). The model revealed an effect of group, *χ* ^2^ (2) = 15.41, *p* < .001. Blind participants mentioned landmarks more than blindfolded (*β* = .421, *SE* = .012, *t* = 3.40, *p* = .003) and sighted (*β* = .452, *SE* = .012, *t* = 3.65, *p* = .002) participants, and there was no difference between blindfolded and sighted participants, *β* = .032, *SE* = .012, *t* = 0.26, *p* = .97.

**Fig. 4:**
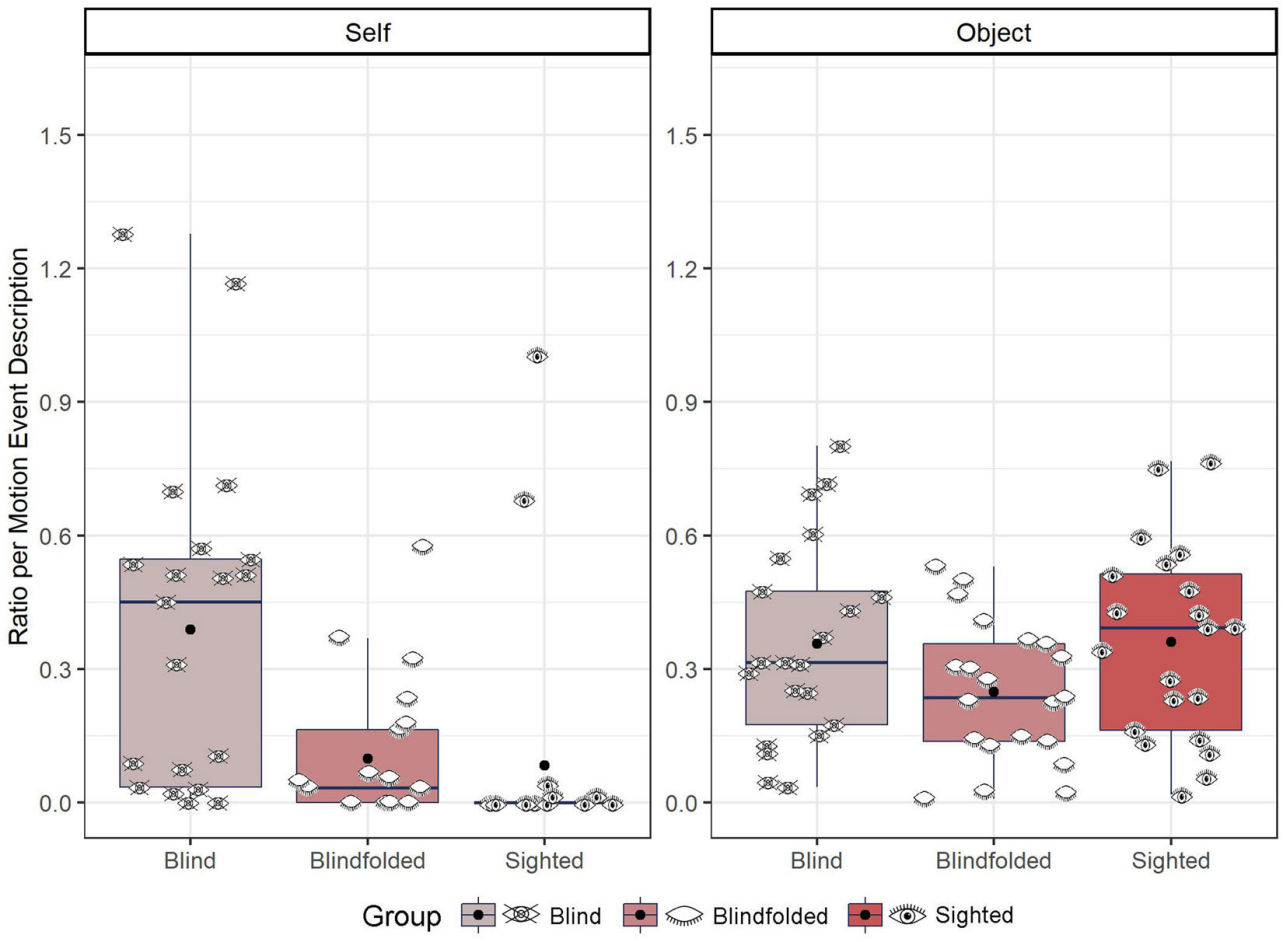
Self and object‐anchored landmarks in speech. Eye icons represent the average ratio for each participant. Black dots represent the group mean.

We further predicted that if blind people rely more on an egocentric frame of reference, they would use more self‐anchored landmarks than blindfolded and sighted participants. In contrast, blindfolded and sighted people would use more object‐anchored landmarks than blind participants. To test this, we calculated the ratio of mention of self‐anchored and object‐anchored landmark per motion event description for each participant and item. Then, we ran an lmer model with the fixed factors of group and landmark reference (object‐ or self‐anchored) using the number of mention of landmark per motion event description as the dependent variable (Fig. 4). The model revealed an effect of group, *χ* ^2^ (2) = 14.98, *p* < .001, showing that blind participants mentioned more landmarks in their speech than non‐blind participants, and an effect of landmark category, *χ* ^2^ (2) = 160.33, *p* < .001, showing that object‐anchored landmarks were mentioned more than self‐anchored landmarks. Yet, the model also revealed an interaction between group and landmark category, *χ* ^2^ (2) = 161.03, *p* < .001. To follow‐up the interaction, we compared the effect of group separately by landmark category. As expected, blind participants referred to self‐anchored landmarks more than blindfolded (*β* = .292, *SE* = .053, *t* = 5.50, *p* < .001) and sighted (*β* = .305, *SE* = .053, *t* = 5.74, *p* < .001) participants, and there was no difference between blindfolded and sighted participants (*β* = .014, *SE* = .053, *t* = 0.25, *p* = .97). But, the groups did not differ in terms of reference to object‐anchored landmarks, all *p*s > .10.

#### 3.1.3 Path and manner use in speech

**Fig. 5:**
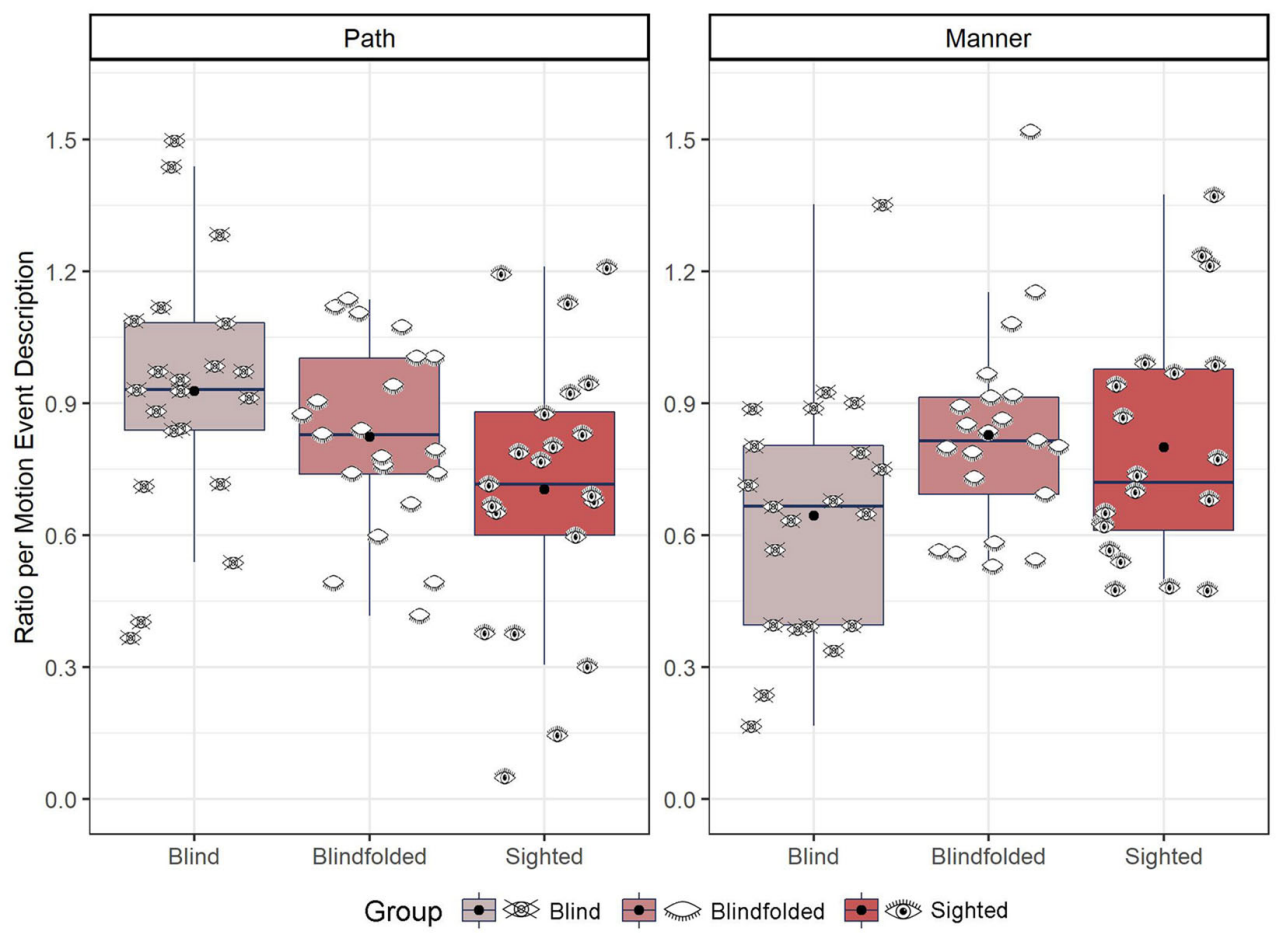
Path and manner in speech. Eye icons represent the average ratio for each participant. Black dots represent the group mean.

Next, we examined whether participants differed in how they expressed path and manner in speech. For this, we calculated the ratio of mention of path and manner per motion event description for each participant and item. We ran an lmer model with the fixed factors of group and type of expression (path vs. manner) and their interaction term using the ratio of mention of path and manner per motion event description as the dependent variable (Fig. 5). The model revealed no effect of group, *χ* ^2^ (2) = 0.68, *p* = .71, no effect of type of expression, *χ* ^2^ (2) = 0.004, *p* = .95, but an interaction between group and type of expression, *χ* ^2^ (2) = 16.31, *p* < .001. To follow‐up the interaction, we used the *emmeans* function to compare the groups for path and manner use separately.

For path, although the interaction was significant in the model, there was no difference in the mention of path between blind and sighted (*β* = .224, *SE* = .103, *z* = 2.18, *p* = .075), blind and blindfolded (*β* = .104, *SE* = .102, *z* = 1.02, *p* = .56), or blindfolded and sighted (*β* = .120, *SE* = .102, *z* = 1.17, *p* = .47). However, the difference between blind and sighted participants (*β* = .224, *SE* = .104, *t* = 2.15, *p* = .033) was significant when we did not use the conservative *p*‐adjustment in *emmeans*: blind participants mentioned path more than sighted participants.

For manner, blind participants mentioned manner less often than sighted (*β* = –.345, *SE* = .104, *t* = –3.32, *p* = .001) but not blindfolded (*β* = –.183, *SE* = .103, *t* = –1.78, *p* = .08) participants. There was no difference between blindfolded and sighted participants *β* = –.162, *SE* = .104, *t* = –1.56, *p* = .12). The interaction between group and type of expression can be seen in Fig. 5.

### 3.2 Gesture

As with speech, we first examined the overall amount of gesture produced by each group, before comparing landmark gestures, and path and manner gestures. As the amount of gesture changes as a function of the rate of motion event descriptions, we first calculated the gesture ratio per motion event description by dividing the total number of gestures by the total number of motion event descriptions. To further investigate what type of gestures participants produced, we calculated the number of pointing gestures referring to localization of landmark (hand and head pointing combined) and iconic (path‐only, manner‐only, and path+manner) gestures per motion event description for each participant and item. For these calculations, total counts of pointing gestures, path‐only, manner‐only, and path+manner gestures were divided by the number of motion event descriptions for each trial. Hand gestures constitute 81.5% of pointing gestures. The data were analyzed in the same way as speech.

#### 3.2.1 Overall gesture rate

We compared the groups in terms of their overall gesture ratio using a one‐way between‐participants ANOVA. There was a significant difference in the gesture ratio between blind (*M* = 0.44, *SD* = 0.48), blindfolded (*M* = 0.82, *SD* = 0.53), and sighted (*M* = 0.69, *SD* = 0.47) participants; *F*(2,60) = 3.18, *p* = .049. A post‐hoc Tukey test showed that blindfolded participants had more gestures than blind participants (*p* = .041), but there was no difference between sighted and blind (*p* = .25) or blindfolded and sighted participants (*p* = .65).

#### 3.2.2 Pointing gestures to landmarks

**Fig. 6:**
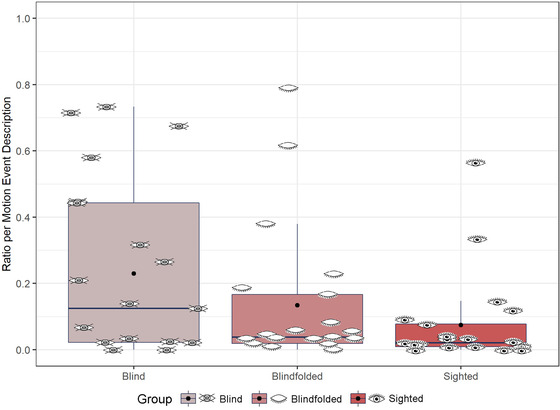
Pointing gestures to landmarks. Eye icons represent the average ratio for each participant. Black dots represent the group mean.

We predicted that if blind participants would use more landmarks in their speech than non‐blind participants, this might be reflected in more pointing gestures to landmarks (Fig. 6), and Section 3.1.2 showed that blind individuals did mention landmarks more often. To test for differences in gesture, we ran an lmer model with the fixed factor of group using the number of pointing gestures per motion event description as the dependent variable. The model revealed a marginal effect of group, *χ* ^2^ (2) = 5.81, *p* = .055. Blind participants produced more pointing gestures than sighted (*β* = .156, *SE* = .064, *z* = 2.45, *p* = .038) but not blindfolded participants (*β* = .095, *SE* = .064, *z* = 1.50, *p* = .29). There was no difference between blindfolded and sighted participants (*β* = .060, *SE* = .064, *z* = 0.95, *p* = .61).

#### 3.2.3 Path and manner gestures

**Fig. 7:**
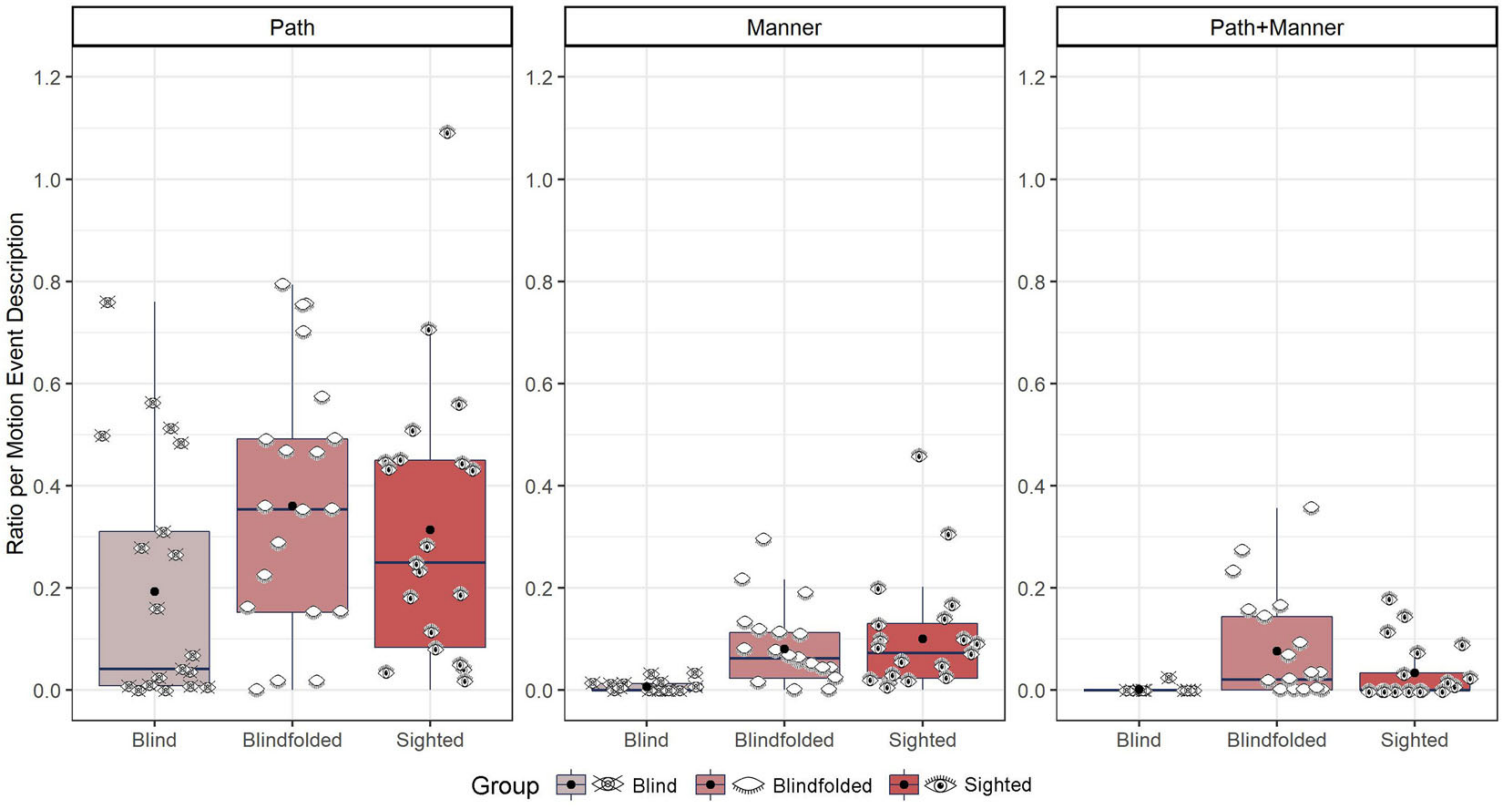
Path and manner gestures for motion event descriptions. Eye icons represent the average ratio for each participant. Black dots represent the group mean.

To compare iconic gestures, we ran an lmer model with the fixed factors of group and type of expression (path‐only, manner‐only, and path+manner) using the ratio of path and manner gestures per motion event description as the dependent variable (Fig. 7). The model revealed an effect of group, *χ* ^2^ (2) = 10.39, *p* = .006, an effect of type of expression, *χ* ^2^ (2) = 1354.7, *p* < .001, and an interaction effect of group and type of expression, *χ* ^2^ (2) = 52.67, *p* < .001.

All groups produced more path‐only gestures than manner‐only (*β* = .227, *SE* = .007, *t* = 30.99, *p* < .001) or path+manner gestures (*β* = .253, *SE* = .007, *t* = 34.54, *p* < .001). To follow‐up the interaction, we used the *emmeans* function to compare the groups for each gesture type separately. Blind participants produced fewer path‐only gestures than blindfolded (*β* = –.167, *SE* = .035, *t* = –4.81, *p* < .001) and sighted (*β* = –.119, *SE* = .035, *t* = –3.41, *p* = .001) participants. Also, blind participants produced fewer manner‐only gestures than blindfolded (*β* = –.074, *SE* = .035, *t* = –2.15, *p* = .037) and sighted participants (*β* = –.096, *SE* = .035, *t* = –2.76, *p* = .008). Blind participants also produced fewer path+manner gestures than blindfolded (*β* = –.075, *SE* = .035, *t* = –2.15, *p* = .036) but not sighted (*β* = –.033, *SE* = .035, *t* = –.94, *p* = .35) participants. There was no difference between blindfolded and sighted participants in terms of path‐only (*β* = .048, *SE* = .035, *t* = 1.39, *p* = .17), manner‐only (*β* = –.022, *SE* = .035, *t* = –.62, *p* = .54), or path+manner (*β* = .042, *SE* = .035, *t* = 1.21, *p* = .23) gestures.

Overall, then, blind participants produced fewer iconic gestures—both path and manner—than blindfolded and sighted participants, but there was no difference between blindfolded and sighted participants.

## 4 Discussion

Our findings point to some similarities, but also notable differences between blind people's multimodal language use and their sighted and blindfolded counterparts. All speakers produced a comparable amount of motion event descriptions in their speech, but differed in how they referred to certain aspects of events. In comparison to non‐blind (both blindfolded and sighted) speakers, blind speakers were more likely to use landmarks and, in particular, more self‐anchored landmarks. In addition, blind speakers tended to talk more about path and less about manner of motion events than sighted speakers. With regard to co‐speech gesture, we observed a similar gesture rate between blind and sighted speakers. However, speakers’ gesture frequency differed depending on the gesture type: blind speakers produced more pointing gestures with landmarks than sighted speakers, but had fewer path and manner gestures than non‐blind speakers (blindfolded and sighted). Even though all speakers’ gesture patterns were consistent with the Turkish motion typology (i.e., path dominant gestures), blind speakers produced fewer iconic gestures than non‐blind speakers overall. We contextualize and discuss each of these points in more detail.

The fact that Turkish blind and non‐blind (blindfolded and sighted) individuals did not differ in the overall amount of verbal descriptions produced is perhaps not surprising given that blind people are good at processing auditory information (e.g., Battal et al., 2020; Gougoux et al., 2004; Röder et al., 1999; Wan et al., 2010). Similarly, we found that co‐speech gesture rates were comparable between blind and sighted individuals, although blind people gestured less than blindfolded speakers. At first glance, this seems partially inconsistent with what has been reported in earlier studies—i.e., blind speakers produce less gesture than sighted speakers (Iverson, 1999; Iverson & Goldin‐Meadow, 1997; Özçalışkan et al., 2016, 2018). However, this apparent contradiction could be because earlier studies focused only on iconic gesture production, whereas the current study examined different gesture types—both pointing and iconic.

Although overall rates of speech and gesture were comparable across groups, there were notable qualitative differences in the verbal and gestural expressions which merit further discussion. For example, blind speakers mentioned landmarks more than non‐blind (blindfolded and sighted) speakers. In particular, when landmarks were mentioned, blind speakers were more likely to refer to them in relation to their own position (e.g., self‐anchored; *from my left*). We also found that blind speakers had more pointing gestures to posited landmarks in gesture space than sighted speakers. Taken together, this is in line with previous studies that find blind people rely more on egocentric than allocentric frames of reference when learning spatial layouts (e.g., Cattaneo & Vecchi, 2011; Iachini et al., 2014; Pasqualotto & Proulx, 2012; Ruggiero et al., 2021). Thus, our results provide further linguistic evidence for the use of an egocentric frame of reference in the spatial language (see also Iverson, 1999; Iverson & Goldin‐Meadow, 1997).

Blind speakers also used more path verbs than sighted speakers. Previous route description studies (Iverson, 1999; Iverson & Goldin‐Meadow, 1997) found that blind people use landmarks on routes and suggest this is because blind people segment paths more in order to make routes more navigable. Although our motion events had single paths (i.e., smaller‐scale in comparison to earlier route description studies with multiple paths), speakers could still segment paths into smaller units by mentioning landmarks more and, thus, utilizing different path verbs in their descriptions of a single event (e.g., *someone came from my side and went away towards the elevator*). So, this path segmentation is a result of more mentions of landmarks (e.g., “from my side” and “towards the elevator”). Together with the increased landmark use, increased mention of path in speech suggests that blindness may enhance sensitivity to paths due to changes in event construal that arise from altered spatial cognition (e.g., Cattaneo & Vecchi, 2011; Lessard et al., 1998; Röder et al., 1999; Voss et al., 2004). At the same time, blind speakers did not differ from blindfolded speakers, suggesting that a temporary lack of vision through blindfolding at encoding can also lead to changes in the encoding of path in motion events.

In contrast to speech, blind speakers used fewer path gestures than non‐blind (blindfolded and sighted) speakers. Even though there was a mismatch in the frequencies of path in speech and path in gesture, speech and gesture type were still coupled with respect to motion event depictions in Turkish—i.e., separated path and manner use in both speech and gesture (e.g., Kita & Özyürek, 2003; Özçalışkan et al., 2016, 2018). The reduced frequency of path gestures from blind speakers could arise for a different reason, however, namely because gesture frequency decreases when paths are more segmented in speech, as suggested by earlier studies (Iverson, 1999; Iverson & Goldin‐Meadow, 1997). This could be because gestures are better suited for holistic than segmented expression due to their visual format (McNeill, 1992; McNeill & Duncan, 2000).

In contrast to path talk, blind speakers mentioned manner less often in speech than sighted speakers. Earlier language comprehension studies have shown that blind and sighted speakers have similar semantic knowledge of action and motion verbs (e.g., Bedny et al., 2008, 2012, 2019), but our findings suggest that semantic knowledge of motion verbs might not be enough to map the sounds of locomotion to manner verbs. In addition, blind speakers had almost no manner gestures except for very few cases where they represented manner of motion bodily—e.g., imitating a person running using the upper body. The lack of manner in the speech and gesture production of blind individuals could be the result of a lack of visual experience; perhaps it is harder to learn manner distinctions from auditory input. However, there is an alternative possibility: Turkish is a verb‐framed language, and sighted Turkish speakers tend to omit manner more often than speakers of satellite‐framed languages, such as English (e.g., Kita & Özyürek, 2003; Özçalışkan et al., 2016, 2018; Slobin, 1996; Talmy, 1985). So, the paucity of manner in the speech and gesture of blind participants could be the result of language statistics, rather than a lack of perceptual access. Further studies could disentangle these possibilities by examining how manner expressions are modulated by both visual experience and language typology, particularly in manner‐dominant languages (i.e., satellite‐framed languages, such as English).

The comparison of blind and blindfolded speakers enabled us to differentiate the effect of momentary lack of vision from the long‐term effect of blindness. Even though blind participants differed from blindfolded participants, there were cases when the blindfolded group was indistinguishable from the blind and sighted groups, while the blind and sighted groups differed from each other (e.g., in the use of path in speech and pointing gestures). This could suggest an additional role of momentary lack of vision in the expression of spatial language (see also Mamus et al., 2019); however, additional research is needed to establish this definitively.

Finally, the gestures of congenitally blind speakers offer fresh insights into multimodal language production theories. Our results showed that both blind and sighted speakers’ gesture patterns were in line with what we would expect considering the typology of a verb‐framed language, i.e., Turkish (e.g., Kita & Özyürek, 2003; Özçalışkan et al., 2016, 2018; Ter Bekke et al., 2022). All speakers gestured more about path than manner of motion. This supports claims that language typology is the determining factor in co‐speech gesture production, even in blind speakers (e.g., Özçalışkan et al., 2016, 2018). Moreover, the alignment between blind people's speech and gesture (i.e., more landmark mentions with more pointing to landmarks and reduced manner mentions with fewer manner gestures) is in line with integration theories of speech and gesture (e.g., Kita & Özyürek, 2003). The fact that blind people had fewer iconic gestures overall than non‐blind people is also in line with theories highlighting the role of visuo‐spatial imagery underlying iconic gesture production (e.g., Hostetter & Alibali, 2008, 2019). Possibly, co‐speech gesture derives partly from language typology and partly from visuo‐spatial imagery (Kita & Özyürek, 2003).

## 5 Conclusion

Theories of embodied cognition propose that multimodal language processes are rooted in sensory and motor experience (Barsalou, 2016; Hostetter & Alibali, 2008; Pouw et al., 2014; Wilson, 2002). There is also substantial evidence that spatial cognition differs between blind and sighted people (Cattaneo & Vecchi, 2011; Lessard et al., 1998; Röder et al., 1999; Ruggiero et al., 2021; Voss et al., 2004). Thus, a lack of visual experience may shape spatial language via altered spatial cognition. In line with this, we find differences in spatial language use in response to auditory motion events experienced by blind and sighted individuals. To disentangle the effects of a lifetime experience of being blind versus the task‐specific effects of experiencing a motion event by sound alone, we included a third condition of sighted individuals who were blindfolded during the task.

Overall, we found that blind people were more likely to mention landmarks, especially those in relation to themselves, than both sighted and blindfolded people. They were also more likely to mention path of motion in speech than sighted people while omitting manner in both speech and gesture. However, based on our current data, we cannot rule out the possibility that blind speakers of a satellite‐framed language may show more resilience in extracting manner information from the sound. While the verbal encoding of path and manner did not differ between blind and blindfolded people, the differences in the gestural encoding of path and manner distinguished blind people from both sighted and blindfolded people. This suggests that beyond merely a temporary lack of sight, a lifetime of blindness changes how these components are represented in gesture. This may be because iconic gestures are more difficult to build upon non‐visual information alone.

Although the current data illustrate differences between blind and sighted people, it remains unclear whether the differences in language use occur because blind people's lifetime of perceptual experience influences their conceptualization of spatial events or because blind people extract event information from auditory input for linguistic expressions differently than sighted people. Further research on blind people's language use is needed to uncover precisely how perceptual experience shapes multimodal language.

Taken together, our study illustrates that a lack of visual experience affects how people encode spatial events for multimodal language production.

### Open Research Badges

This article has earned Open Data and Open Materials badges. Data and materials are available at https://osf.io/qsr7j/.
